# Sexual dimorphisms in genetic loci linked to body fat distribution

**DOI:** 10.1042/BSR20160184

**Published:** 2017-02-03

**Authors:** Sara L. Pulit, Tugce Karaderi, Cecilia M. Lindgren

**Affiliations:** 1 Department of Neurology, Brain Center Rudolf Magnus, University Medical Center Utrecht, Utrecht, The Netherlands; 2 Department of Biological Sciences, Faculty of Arts and Sciences, Eastern Mediterranean University, Famagusta, Cyprus; 3 Big Data Institute, Li Ka Shing Centre for Health Information and Discovery, University of Oxford, Oxford, U.K.; 4 Wellcome Trust Centre for Human Genetics, Nuffield Department of Medicine, University of Oxford, Oxford, U.K.

**Keywords:** genetics, obesity, sex

## Abstract

Obesity is a chronic condition associated with increased morbidity and mortality and is a risk factor for a number of other diseases including type 2 diabetes and cardiovascular disease. Obesity confers an enormous, costly burden on both individuals and public health more broadly. Body fat distribution is a heritable trait and a well-established predictor of adverse metabolic outcomes. Body fat distribution is distinct from overall obesity in measurement, but studies of body fat distribution can yield insights into the risk factors for and causes of overall obesity. Sexual dimorphism in body fat distribution is present throughout life. Though sexual dimorphism is subtle in early stages of life, it is attenuated in puberty and during menopause. This phenomenon could be, at least in part, due to the influence of sex hormones on the trait. Findings from recent large genome-wide association studies (GWAS) for various measures of body fat distribution (including waist-to-hip ratio, hip or waist circumference, trunk fat percentage and the ratio of android and gynoid fat percentage) emphasize the strong sexual dimorphism in the genetic regulation of fat distribution traits. Importantly, sexual dimorphism is not observed for overall obesity (as assessed by body mass index or total fat percentage). Notably, the genetic loci associated with body fat distribution, which show sexual dimorphism, are located near genes that are expressed in adipose tissues and/or adipose cells. Considering the epidemiological and genetic evidence, sexual dimorphism is a prominent feature of body fat distribution. Research that specifically focuses on sexual dimorphism in fat distribution can provide novel insights into human physiology and into the development of obesity and its comorbidities, as well as yield biological clues that will aid in the improvement of disease prevention and treatment.

## Introduction

### Sexual dimorphism in health and disease

Sexual dimorphism is the differentiation between the male and female sexes in both morphology and phenotype. So-called primary sex traits, such as the male and female sex organs and reproductive traits, are evidently sexually dimorphic. Other sexually dimorphic characteristics in animals are visually striking: the elaborate tail of the male peacock, the male lion’s mane and the increased body size of some female spiders [1]. Animal models have helped reveal additional biological sexually dimorphic characteristics, including sex-specific neural circuitry in flies [2,3] and sex-specific gene expression in mammals [2,4].

Despite centuries of observation of such sexually dimorphic characteristics and features, little is understood about the aetiology of sexually dimorphic traits. The sex chromosomes are an obvious first culprit in identifying biological drivers of sexual dimorphism in animals. Males can exhibit recessive diseases carried on the X chromosome; an example is red-green colour blindness, an X-linked disease that is one of the most prevalent sexually dimorphic traits (prevalence approximately 5% in males and <1% in females) [5]. Female carriers of recessive X-linked diseases are not necessarily unaffected by the disease allele; these female carriers of X-linked disease mutations can have altered X-chromosome inactivation [2] and can also demonstrate diminished severity of disease [2,6].

The X chromosome is not the only biological regulator of sexually dimorphic characteristics. The role of the autosomal genome is also crucial when considering the genetic architecture of sexually dimorphic traits. On a molecular level, several species have been shown to have sexually dimorphic gene expression across the genome and across tissues and organs [2]. Additionally, a recent investigation of common genetic variants (or single nucleotide polymorphisms, called SNPs) [7] indicated that many genes across the human genome are undergoing sex-specific selection, selection that results in biased gene expression between males and females; this observation was also replicated in data drawn from flies [8], suggesting a mechanism conserved across species. It remains difficult, however, to pinpoint precisely when this selection occurs and its downstream phenotypic effect on both anthropometric traits and on health and diseases more generally.

### Body fat distribution and sexual dimorphism

Beyond the immediate benefit of enabling sexual reproduction, sexually dimorphic traits may bestow their carriers with particular advantages. For example, it is hypothesized that “ornamental” [1,9] males may make more advantageous mates, as they are more likely to provide particular benefits such as physical protection or care to a pregnant partner [1,9]. Though sexual dimorphism may confer health and reproductive benefits, it has also been observed in a wide range of common diseases in animals (both human and nonhuman) that are complex in aetiology, including both an environmental and a biological component. In children, for example, autism is more common in boys, whereas scoliosis is more common in girls [2]. Sexual dimorphism persists in later onset diseases as well: Parkinson’s disease, schizophrenia [2] and amyotrophic lateral sclerosis are all more common in men [10], whereas women are at a higher risk of autoimmune diseases, major depression [2] and hypertension [11].

Body composition and fat distribution are also complex traits that have been observed to be sexually dimorphic in both humans and nonhumans, particularly in (nonhuman) animal models [12]. There are numerous ways to measure body composition and fat distribution including, but not limited to: body mass index (BMI), height, weight, waist-to-hip ratio (WHR), waist circumference (WC) and hip circumference (often denoted as simply “HIP”). Many of these traits are closely linked. For example, BMI or overall obesity is significantly correlated with body fat distribution (Pearon's r~0.60) [13]. Both WC and WHR are significantly correlated with MRI measurement of central adiposity, accepted as the “gold standard” in fat measurement. WC correlates modestly to visceral fat as measured by MRI (r^2^ = 0.6), as does WHR (r^2^ = 0.5) [13]. BMI, too, correlates with total adipose tissue in an individual, but it cannot capture measures of different types of fat [14].

**Figure 1:**
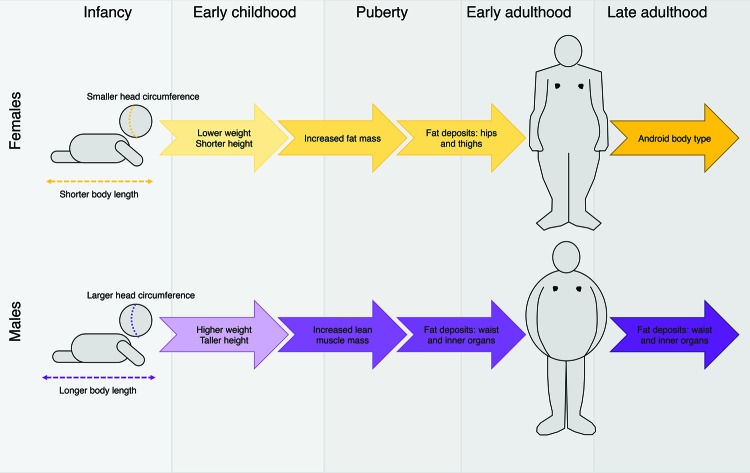
Sexual dimorphism in body shape and fat distribution over a human lifespan Sexual dimorphism in body shape and fat distribution can be observed from birth: male infants are typically born with larger head circumference and longer body length than females. In early childhood development, males remain typically heavier and taller than females. At puberty, sexual dimorphism is more marked. Females gain in fat mass whereas males gain in lean muscle mass. In early adulthood, fat deposits in women typically collect around the hips and thighs whereas in men, fat deposits collect around the inner organs and waist. After menopause in women, the body shape often shifts to a more android (square) body shape.

For studies that aim to understand the biology of fat distribution, WHR has emerged as a particularly powerful phenotype. WHR is a measure of fat distribution that in a single continuous phenotype captures two distinct components: first, it indicates the amount of visceral fat in an individual [15], fat that is typically considered to be metabolically adverse; second, it captures gluteal fat, associated with protective outcomes (i.e. mitigated risk) for type 2 diabetes (T2D) and cardiovascular disease [16,17]. Identifying anthropometric measures like WHR that can capture different types of fat is the key for understanding sexual dimorphism in fat distribution; women tend to have more subcutaneous adipose tissue (SAT) and men more visceral adipose tissue (VAT) [18] and genetic association studies have revealed polymorphisms specific to these tissues. Such findings emphasize the distinct role for biological differences that underpin fat distribution in males and females [19]. Finally, both sexes tend to have different fat depots, which affect body shape (e.g. WHR; Figure 1) but not necessarily overall BMI, further demonstrating that alternative anthropometric traits (rather than BMI) are the key for genetic association studies looking to further elucidate the genetic architecture of obesity in humans.

In addition to being a metric for body composition, distribution of adipose tissue is a phenotype closely linked to disease [20,21]. Body fat distribution is a key measure in evaluating risk for adverse cardiometabolic outcomes, a risk that is in fact distinct from the effect that overall obesity has on disease [22]. Epidemiological studies of body fat distribution (measured proximally by WHR and other anthropometric measures) have revealed an association with increased risk of cardiovascular disease, T2D, stroke, hypertension and a host of other common diseases [19,23]. The worsening obesity epidemic in the United States and around the globe has dramatically increased the incidence of these diseases. Risk of these diseases affects not only adult-age individuals, but younger ones as well. As an example, obesity is a primary risk factor for gestational diabetes mellitus (GDM) [24], which increases the risk of death for the mother and the child, increases risk of cardiometabolic diseases as well as increased risk of obesity later in life [25]. In addition to increasing risk of disease, obesity can also adversely affect fertility and reproduction. Obesity can increase the risk of infertility, miscarriage and complications during pregnancy [26,27]. Notably, infertility in particular is associated not only with obesity, but also with being underweight. This “U-shaped” risk curve is observable in both males and females [28] and emphasizes the importance of studying fat distribution (as opposed to strictly excess fat) more generally.

## Evidence for sexual dimorphism in fat distribution

### Sexual dimorphism in fat distribution: nonhumans

The evidence for sexual dimorphism in fat distribution spans decades of epidemiological and biological data collection and observations in a variety of species. Microarray experiments in mice have demonstrated that adipose tissue mass, function and distribution are regulated by networks of sexually dimorphic genes [29,30] and that developmental genes (defined as genes in the Genes Ontology Biological Processes database annotated for search terms including “organogenesis” and “embryonic development” [31]) are likely to contribute to the underpinnings of obesity and fat distribution [31]. In a study of mice kept on a high-fat diet for 12 weeks, microarray analysis indicated that >1,000 genes in intra-abdominal and gonadal adipose tissue were differentially expressed between male and female mice [32].

### Sexual dimorphism in fat distribution: humans

The epidemiological evidence for sexual dimorphism in humans is extensive. Sexual dimorphism in body composition is already evident in infancy: males tend to be heavier than females at birth, as well as have longer bodies and larger head circumference (Figure 1) [12]. In early childhood, differences between the sexes is evident in standard anthropometric measures such as height and weight, and also in specific fat measures, such as lean mass and total fat mass [33]. Although sexually dimorphic characteristics are more subtle in early life, sexual dimorphism in body composition and fat distribution becomes more distinct in adolescence, when males grow taller compared to females and acquire more muscle mass (Figure 1) [30]. Additionally, although both sexes generally tend to lose mass in earlier years (approximately between ages 1 and 6), during puberty, females begin to accumulate more overall fat mass whereas males accumulate lean muscle (Figure 1) [12].

By early adulthood, sexual dimorphism in fat distribution is highly evident. Female body type tends to be that of the “hourglass” or “pear” shape, with fat deposited most frequently around the hips and thighs (Figure 1) [34]. This shape results from accumulation of subcutaneous fat in women that is preferentially deposited around these regions of the body [35,36]. Pregnancy can emphasize sexually dimorphic fat distribution; an increase in body fat often occurs with pregnancy and, postpartum, there can be an increase in fat deposited around the abdomen as well [12,37,38]. In contrast, men are typically broad-shouldered and narrow-waisted [12], but often develop fat accumulation around the middle region later in life. Males tend to accumulate more visceral fat, which collects around the inner organs and thus appears (outwardly) to deposit most frequently around the waist (Figure 1) [39].

Evidence of sexually dimorphic fat distribution is observable in humans later in life as well. As an example in women, body shape and composition often changes during menopause, when the hourglass shape in women can shift towards a more android body type, which includes increased fat deposition around the abdomen [30]. This shift suggests a role of sex hormones in sexual dimorphism of fat, and in particular the sexually dimorphic expression of hormones between men and women [12,30]; the biological mechanism for this observation, however, is still unknown.

## The heritability of sexual dimorphism in fat distribution

Since well before the mapping of the human genome, epidemiologists used families (and particularly twins) to estimate the heritability of fat distribution in humans. In 1987, Bouchard and colleagues [40] used three measures – BMI, skinfolds and underwater body weight – to estimate the genetic and environmental components of body composition. Their estimates provided some of the first empirical evidence that body mass and composition are, in part, biologically determined. They estimated the genetic heritability of BMI, subcutaneous fat and other fat measures, to be approximately 30% [40]. In 1990, a study of 265 white male twins estimated the heritabilities of WHR at 31% [41]. More recent estimates remain quite similar to those previously reported: overall heritability of BMI is broadly estimated from 41 to 90% in twin studies and population-level analysis, though estimates can vary greatly depending on the studied sample [42,43]. Heritability of fat distribution phenotypes WHR and WC are estimated to be approximately 31 and 39% respectively [43].

**Figure 2:**
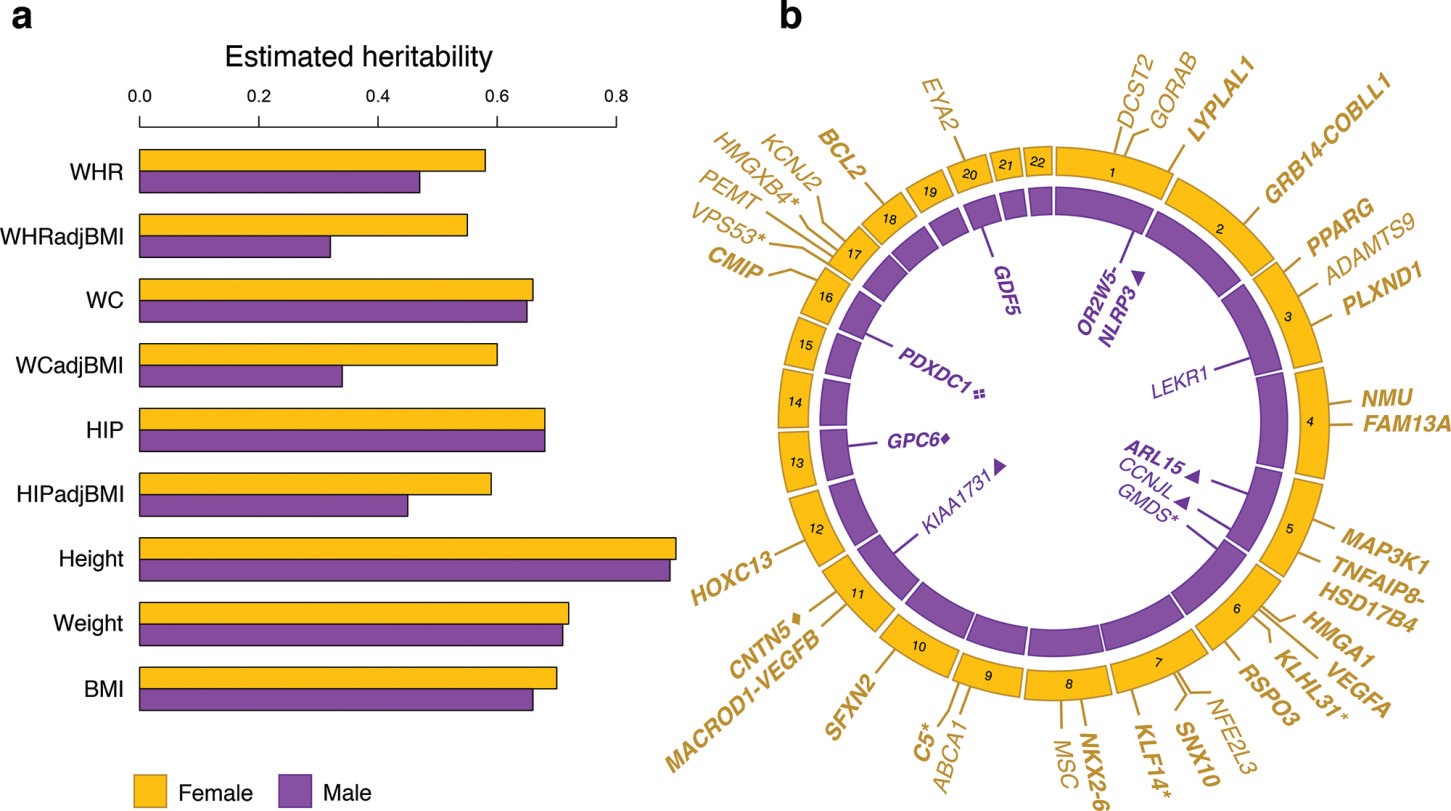
The genetics of sexually dimorphic traits that measure fat distribution (**a**) A number of anthropometric traits, such as height, weight, BMI, HIP and WC have been demonstrated to have similar genetic heritability estimates between males and females. Additional traits, however, have been shown to be sexually dimorphic. In particular, heritability calculations indicate that WHR, WHRadjBMI, WCadjBMI and HIPadjBMI are sexually dimorphic; the heritability estimates for these traits are systematically higher in females (yellow) compared with males (purple). These findings suggest a stronger genetic contribution to these traits in females, and biological mechanisms that are different or differentially expressed between males and females. (**b**) Several sexually dimorphic loci associated with anthropometric and fat distribution traits have been implicated through genome-wide association studies (GWAS). Here, we annotate the chromosomal position of loci that appear at genome-wide significance in female-only (yellow) or male-only (purple) analyses. Loci that have significantly different *P*-values in females compared with males (or vice versa) appear in bold. All loci annotated here were discovered in WHRadjBMI except for loci discovered in HIP (four-piece diamond), HIPadjBMI (asterisks), WCadjBMI (triangles) and WHR (solid diamonds). Note that the genes annotated here reside closest to the associated SNP but are not necessarily causal. Data from the figure were drawn from Tables 1 and 2 in [20].

Interestingly, heritability estimates for traits linked to body fat distribution are not necessarily consistent across the sexes. A recent large-scale meta-analysis of common genetic variants in humans provided heritability estimates for a number of anthropometric traits, including traits related to body fat distribution [20]. The study found that some anthropometric traits, including BMI, weight and height, show minimal or no distinctness when measured in only males or females (Figure 2a) [20]. However, WHR and WC are accepted as better proxies for understanding fat distribution. The study found that in these fat distribution-related traits (WHR, WHR adjusted for BMI (WHRadjBMI), HIP adjusted for BMI (HIPadjBMI) and WC adjusted for BMI (WCadjBMI)), the heritability calculations in males and females were quite distinct (Figure 2) [20]. An additional study of metabolic-related traits in the Dutch Twins Registry further demonstrated sexual dimorphism in fat distribution traits: in younger participants in the study (age <42 years) the heritability of WC and WHR was approximately 30–35% in males, in contrast with 45–50% heritability in females [43]. An additional study of female twins estimated the heritability of WHR to be 36–61% and the heritability of WC to be higher, at 72–82% [44]. These findings indicate that some traits (e.g., BMI) have highly overlapping genetic architectures between males and females, whereas other traits (e.g. WHRadjBMI and HIPadjBMI) have somewhat (though not entirely) distinct genetic architectures in males and females. Thus, there are likely biological pathways unique to or differentially expressed in males and females that contribute to fat distribution. The sexual dimorphism in these heritabilities also indicates that risk for diseases associated with obesity or fat distribution may also have biological drivers that are specific or differentially regulated in one sex compared with the other.

Noticeable in the data we present here, and in many heritability calculations, the estimates of genetic heritability in complex traits can be broad and imprecise. Biases, including sample ascertainment and assumptions regarding shared environment, flaws in data interpretation and limited sample size can all influence the precision of heritability estimates [45]. Nevertheless, various lines of epidemiologic and genetic evidence point to potentially distinct biological architectures in traits related to body fat composition between the sexes. Further, sexually dimorphic biological mechanisms have already been observed in males and females, providing an additional layer of evidence for sex-specific effects on body fat distribution. For example, studies of mRNA and miRNA expression in humans have revealed sexually dimorphic patterns in tissue of the abdomen as well as gluteal adipose [46,47].

## Sexual dimorphism in fat distribution: the genetic evidence

### Genome-wide association studies in fat distribution traits

Genome-wide association studies (GWAS) have been a powerful tool in identifying common genetic variants (SNPs with a minor allele frequency (MAF) > 1%) associated with common, complex diseases and traits. By interrogating SNPs across the span of the human genome and examining frequency differences in SNPs that are associated with phenotypic outcome, GWAS have yielded hundreds of loci associated with thousands of traits [48]. Consortia centred around fat distribution traits including WC, HIP and WHR have assembled genotyping data in thousands of samples and revealed over 100 loci that harbour common-variant associations to these traits [13,14,20,21]. A number of large-scale GWAS have sought to identify common SNPs influencing overall obesity through BMI [49–51], the largest and most recent of which analysed more than 339,000 individuals and identified 97 loci associated with the trait [21]. Other GWAS have focused on body fat percentage (BF%) as a phenotypic outcome. A GWAS of BF% in >100,000 individuals revealed 12 loci reaching genome-wide significance (*P* < 5 × 10^−8^) [52]. Several of these signals overlapped signals found in GWAS of BMI, but conferred stronger effects in BF%, suggesting the importance of their role in adiposity specifically (as opposed to BMI, which measures both fat and lean mass) [52]. A GWAS of WHRadjBMI in >224,000 samples revealed 49 significantly associated loci, many of which contained genes expressed in adipose tissue [20]. GWAS have also revealed potential overlapping genetic architectures between monogenic and population-level forms of obesity. Although mutations in the *MC4R* gene, for example, are the leading cause of monogenic obesity in humans, this locus has also revealed through GWAS to contain common, BMI-increasing polymorphisms segregating in population-representative adult samples [53].

### Sexually dimorphic loci discovered through GWAS and biological insights

Beyond implicating key risk loci, GWAS have been helpful in highlighting sexually dimorphic loci in fat distribution traits. Of the 49 associated loci discovered in the most recent WHRadjBMI GWAS, 20 of them (approximately 40%) showed significant sexual heterogeneity [20]. Incredibly, 19 of these 20 dimorphic loci conferred a stronger effect on women as compared with men (Figure 2b) [20]. An intricate sex-specific genetic architecture was discovered at two loci, *HOXC6–HOXC13* and *TBX15–WARS2–SPAG17*. Specifically, conditional analysis in these regions revealed female- and male-specific signals. Of the three independent associations in the *HOXC6–HOXC13* region, one was found to be female-specific (rs1443512, *P*_conditional_ = 1.1 × 10^−14^); in contrast, in the *TBX15–WARS2–SPAG17* region, one of the four independent signals was male-specific (rs1106529, *P*_conditional_ = 4.8 × 10^−9^) [20]. These observations suggest potentially distinct biological mechanism between the sexes as well as common mechanisms which might be influenced by different variants (i.e. genetic heterogeneity). These findings affirm the hypothesis that the sexual dimorphism of fat distribution is at least partially inherited and biologically regulated.

A small number of GWAS studies have focused specifically on uncovering sexually dimorphic loci associated with fat distribution. Only analyses in waist-related phenotypes (as opposed to other anthropometric measures) revealed significant results. In an analysis of 270,000 individuals, seven loci showed genome-wide significant sexual dimorphism: near growth factor receptor-bound protein 14 (*GRB14*)/*COBLL1*,* LYPLAL1*/*SLC30A10, VEGFA, ADAMTS9, MAP3K1, HSD17B4 and PPARG* (Figure 2b) [30]*.* The loci demonstrated to have a stronger effect in women contained a number of genes that are known to be associated with lipid traits or insulin resistance [14,20,30]. One locus (index SNP rs10195252, *P =* 5.9 × 10^−15^) contains the gene *GRB14* [20], which encodes a protein necessary for insulin signalling regulation. The implication of female-specific associations in *GRB14* is not new; additional variants in this region have been discovered previously in WHRadjBMI, and analyses in a number of metabolic phenotypes including blood lipids, SAT and T2D, have also revealed associated variants [14,30,54,55]. Additionally, the sexually dimorphic signal at *GRB14* replicated in a study of African-ancestry individuals [23], suggesting that the signal is constant across populations, as has been true for GWAS findings in other diseases [56]. Animal models have added additional empirical evidence for the potential role of *GRB14* in obesity: specifically, an analysis of *GRB14* expression in adipose tissue found a negative correlation between insulin sensitivity and gene expression [57] in both rodents and humans, and Grb14 knockout mice showed better control of glucose levels (called glucose homoeostasis) [58]. Collectively, the genetic and biological evidence indicate that *GRB14* has a key role in adipose distribution, and understanding the biological role of *GRB14* may yield insights into potential therapeutics used to treat the rapidly growing obesity epidemic.

In addition, the gene *ADAMTS9*, which lies in a locus discovered through GWAS of WHRadjBMI, contains variants that have a stronger effect in women than in men (rs2371767, *P*_men_ = 0.035 and *P*_women_ = 1.2 × 10^−25^ [20]). *ADAMTS9* is a member of the ADAMTS (a disintegrin and metalloproteinase with thrombospondin motifs) family of proteins. These proteins serve a number of roles, including controlling organ maturation and development as well as inhibiting blood vessel formation [59]. Additional GWAS have revealed more information about the potential role for *ADAMTS9*: a variant in *ADAMTS9* (rs6795735, MAF = 59.4%, linkage disequilibrium (LD) to WHRadjBMI SNP rs2371767 = 0.31, HapMap 2 samples of Northern and Western European ancestry living in Utah (the CEU population) [60]) was also found to be nominally associated with decreased high-density lipoprotein (HDL) (rs6795735, *Z*-score = –2.5, *P =* 0.01) and T2D risk (rs6795735, OR = 1.12, *P* = 0.002) through GWAS, but not with BMI [14,61].

### The lack of sexually dimorphic loci in body fat percentage and BMI

Some GWAS of overall obesity have revealed a notable lack of sexual dimorphism. For example, of the four loci discovered by the recently discussed GWAS in BF% (in or near *COBLL1/GRB14, IGF2BP1, PLA2G6, CRTC1*) [52] and in the most recent GWAS of obesity as measured by BMI [21] only a very small number of these overall obesity loci exhibited sexual dimorphism (approximately 3–5%) [21,52]. There are several potential explanations for the apparent lack of sexual dimorphism in BMI and BF% loci compared with WHR. First, biological pathways for fat distribution traits WHR and WC are likely to be quite distinct from BMI. Analysis of the loci implicated by the latest GWAS in BMI revealed a major neuronal component underpinning the trait [21]. In contrast, WHR and WC loci revealed through GWAS have revealed that these traits are more likely to be influenced by early development and/or differentiation of adipocytes [20,21]. These findings again point to a striking difference across the many facets of obesity and raise the possibility that different organs are differentially susceptible for sexually dimorphic effects. Further, these findings reflect important and fundamental physiological differences between men and women (discussed below).

## Discussion

Understanding the underlying biological mechanisms and downstream effects of sexually dimorphic associations with body fat distribution is a crucial step in understanding disease risk and pathogenesis. Further, unravelling the genetic underpinnings of disease and the related sexual dimorphism, may help reveal therapeutic targets or diagnostically relevant mechanisms used to predict or treat obesity in men or women specifically. One such example is *PPARG*, a gene found to be associated with T2D and monogenic forms of severe obesity and severe digenic insulin resistance [62,63]. An SNP in *PPARG* associated with WHRadjBMI exhibits sexual dimorphism, with a significantly stronger effect in women (β_women_ = 0.035, β_men_ = 0.005) [20]. Additionally, a drug trial in PPARG-agonist therapy revealed sexual dimorphism in the response of patients with T2D, indicating that insulin resistance may have (partially) distinct mechanisms in men and women [64]. The findings at *PPARG*, and potentially additional findings at other sexually dimorphic loci revealed through ongoing functional work, may help in our movement towards precision medicine. Clinical trials for obesity treatments or treatments of obesity-related disease, for example, may be improved by stratifying on sex.

Very few GWAS (or large-scale epidemiologic studies) of complex traits and common diseases have explicitly and thoroughly investigated the potential role for sexual dimorphism in the underlying biology of disease. This phenomenon is likely due in part to statistical power; performing sex-specific analyses require reducing the sample size of a study by half, thus also reducing statistical power to reveal an association of subtle effect [65], typical for common-variant associations discovered in common disease [56]. Large-scale consortia and biobanking [66] have now enabled GWAS of common diseases at an unprecedented scale; the number of samples worldwide that have undergone genotyping using SNP arrays now number into millions [21,56,67]. Though sex-specific analyses will still require a loss in power compared with analysing both sexes jointly, this amassing of samples now enables us to investigate sexual dimorphism in obesity and related traits in samples numbering in tens or hundreds of thousands. Such studies may reveal additional sexually dimorphic risk loci associated with not only obesity, but to an array of obesity-related traits and diseases such as cardiovascular disease [22], metabolic diseases [17], schizophrenia [21], fertility [26], irritable bowel syndrome [21] and Alzheimer’s disease [21,27].

Further, genetics and genomics will remain powerful approaches in understanding and treating diseases. Additional GWAS that focus on sex-specific risk can reveal biological information beyond newly discovered risk loci. A number of publicly available software and resources are helping to identify "credible sets" of SNPs in GWAS loci that are most likely to drive observed association signals [68–70], integrate expression information to identify expression quantificative trait loci (eQTLs) [71] and include genome-folding information to disentangle the role of regulatory variation [72]. These tools, in combination with improved functional annotation of the genome [73–75] and increasingly dense sequencing-based imputation reference panels [7,76], will help further advance our understanding of common disease risk from associated locus to biological mechanism. Additionally, breakthroughs in single-cell sequencing [77], RNA sequencing [78,79], and gene editing [80] allow for tissue-specific investigations of gene variants or changes in expression at genes or variants associated with disease. This massive collection and integration of ‘omics’ data will help in completing our understanding of gene networks or regulatory mechanisms that contribute to disease risk or progression, and allow us to understand disease severity in cell, tissue and developmental stage specific settings.

As the obesity epidemic has now reached global proportions, fat distribution and obesity are worthy of scientific study more than ever. The epidemic brings with it an increased prevalence of diseases that are epidemiologically and biologically linked to obesity, such as cardiovascular and metabolic diseases; these diseases are often chronic, can be fatal, and create a profound burden in terms of diminished quality of life, societal cost (e.g., lost work productivity) and healthcare-related monetary costs, which amounts to billions of dollars every year in the United States and elsewhere [81,82]. Due to the environmental components that influence obesity, such as diet and exercise, information on how to curb the epidemic is often drawn from a wide range of sources, including incomplete or biased information. Diet studies, for example, typically rely on survey information, which can be inaccurate or incomplete. Exercise studies are heavily biased towards examining male participants; this bias greatly limits our understanding of the effect of exercise on obesity in women [83], even though women are equally affected by the epidemic [84]. Given the various sources of information around obesity and its treatment and the increasing number of individuals affected by the disease, obesity deseves not only our attention but the utmost scientific rigor as well. Sexual dimorphism and its role in fat distribution, made clear by epidemiologic and genetic studies, will be a key part of unravelling the biological risk factors of obesity in a rigorous and complete manner. In particular, further disentangling the sexual dimorphism at loci associated with fat distribution, as well as designing studies of obesity that explicitly focus on females or males only, will yield insights into the biological signatures of disease that are sex-specific. Such information will improve precision medicine, inform sex-stratified clinical trials, and help in moving us towards treating and ultimately stemming the obesity epidemic worldwide.

## Abbreviations

BF%: body fat percentage
BMI: body mass index
CEU: central europe
GRB14: growth factor receptor-bound protein 14
GWAS: genome-wide association studies
HDL: high-density lipoprotein
HIP: hip circumference
HIPadjBMI: HIP adjusted for BMI
LD: linkage disequilibrium
MAF: minor allele frequency
SAT: subcutaneous adipose tissue
SNP: single nucleotide polymorphism
T2D: Type 2 diabetes
WC: waist circumference
WCadjBMI: WC adjusted for BMI
WHR: waist-to-hip ratio
WHRadjBMI: WHR adjusted for BMI

## References

[1] Williams T.M. and Carroll S.B. (2009) Genetic and molecular insights into the development and evolution of sexual dimorphism. Nat. Rev. Genet. 10, 797–804 19834484 10.1038/nrg2687

[2] Ober C., Loisel D.A. and Gilad Y. (2008) Sex-specific genetic architecture of human disease. Nat. Rev. Genet. 9, 911–922 19002143 10.1038/nrg2415 PMC2694620

[3] Kimura K.-I., Ote M., Tazawa T. and Yamamoto D. (2005) Fruitless specifies sexually dimorphic neural circuitry in the *Drosophila* brain. Nature 438, 229–233 16281036 10.1038/nature04229

[4] Rinn J.L. and Snyder M. (2005) Sexual dimorphism in mammalian gene expression. Trends Genet. 21, 298–305 15851067 10.1016/j.tig.2005.03.005

[5] Birch J. (2012) Worldwide prevalence of red-green color deficiency. J. Opt. Soc. Am. A Opt. Image Sci. Vis. 29, 313–320 22472762 10.1364/JOSAA.29.000313

[6] Dobyns W.B. (2006) The pattern of inheritance of X-linked traits is not dominant or recessive, just X-linked. Acta Paediatr. Suppl. 95, 11–15 16720459 10.1111/j.1651-2227.2006.tb02383.x

[7] 1000 Genomes Project Consortium, Auton A., Brooks L.D., Durbin R.M., Garrison E.P., Kang H.M. (2015) A global reference for human genetic variation. Nature 526, 68–74 26432245 10.1038/nature15393 PMC4750478

[8] Cheng C. and Kirkpatrick M. (2016) Sex-specific selection and sex-biased gene expression in humans and flies. PLoS Genet. 12, e1006170 27658217 10.1371/journal.pgen.1006170 PMC5033347

[9] Andersson M.B. Sexual selection Princeton University Press (1994)

[10] Nelson LM. (1995) Epidemiology of ALS. Clin. Neurosci. 3, 327–331 9021253

[11] Hajjar I. and Kotchen T.A. (2003) Trends in prevalence, awareness, treatment, and control of hypertension in the United States, 1988-2000. JAMA 290, 199–206 12851274 10.1001/jama.290.2.199

[12] Wells J.C. (2007) Sexual dimorphism of body composition. Best Pract. Res. Clin. Endocrinol. Metab. 21, 415–430 17875489 10.1016/j.beem.2007.04.007

[13] Lindgren C.M., Heid I.M., Randall J.C., Lamina C., Steinthorsdottir V., Qi L. (2009) Genome-wide association scan meta-analysis identifies three loci influencing adiposity and fat distribution. PLoS Genet. 5, e1000508 19557161 10.1371/journal.pgen.1000508 PMC2695778

[14] Shuster A., Patlas M., Pinthus J.H. and Mourtzakis M. (2012) The clinical importance of visceral adiposity: a critical review of methods for visceral adipose tissue analysis. Br. J. Radiol. 85, 1–10 21937614 10.1259/bjr/38447238 PMC3473928

[15] Heid I.M., Jackson A.U., Randall J.C., Winkler T.W., Qi L., Steinthorsdottir V. (2010) Meta-analysis identifies 13 new loci associated with waist-hip ratio and reveals sexual dimorphism in the genetic basis of fat distribution. Nat. Genet. 42, 949–960 20935629 10.1038/ng.685 PMC3000924

[16] Manolopoulous K.N., Karpe F. and Frayn K.N. (2010) Gluteofemoral body fat as a determinant of metabolic health.. Int. J. Obes. (Lond.) 34, 949–959 20065965 10.1038/ijo.2009.286

[17] Shuster A., Patlas M., Pinthus J.H. and Mourtzakis M. (2012) The clinical importance of visceral adiposity: a critical review of methods for visceral adipose tissue analysis. Br. J. Radiol. 85, 1–10 21937614 10.1259/bjr/38447238 PMC3473928

[18] Fox C.S., Massaro J.M., Hoffmann U., Pou K.M., Maurovich-Horvat P., Liu C.Y. (2007) Abdominal visceral and subcutaneous adipose tissue compartments: association with metabolic risk factors in the Framingham Heart Study. Circulation 116, 39–48 17576866 10.1161/CIRCULATIONAHA.106.675355

[19] Fox C.S., Liu Y., White C.C., Feitosa M., Smith A.V., Heard-Costa N. (2012) Genome-wide association for abdominal subcutaneous and visceral adipose reveals a novel locus for visceral fat in women. PLoS Genet. 8, e1002695 22589738 10.1371/journal.pgen.1002695 PMC3349734

[20] Shungin D., Winkler T.W., Croteau-Chonka D.C., Ferreira T., Locke A.E., Mägi R. (2015) New genetic loci link adipose and insulin biology to body fat distribution. Nature 518, 187–196 25673412 10.1038/nature14132 PMC4338562

[21] Locke A.E., Kahali B., Berndt S.I., Justice A.E., Pers T.H., Day F.R. (2015) Genetic studies of body mass index yield new insights for obesity biology. Nature 518, 197–206 25673413 10.1038/nature14177 PMC4382211

[22] Pischon T., Boeing H., Hoffmann K., Bergmann M., Schulze M.B., Overvad K. (2008) General and abdominal adiposity and risk of death in Europe. N. Engl. J. Med. 359, 2105–2120 19005195 10.1056/NEJMoa0801891

[23] Liu C.-T., Monda K.L., Taylor K.C., Lange L., Demerath E.W., Palmas W. (2013) Genome-wide association of body fat distribution in African ancestry populations suggests new loci. PLoS Genet. 9, e1003681 23966867 10.1371/journal.pgen.1003681 PMC3744443

[24] Kjos S.L. and Buchanan T.A. (1999) Gestational diabetes mellitus. N. Engl. J. Med. 341, 1749–1756 10580075 10.1056/NEJM199912023412307

[25] Clausen T.D., Mathiesen E.R., Hansen T., Pedersen O., Jensen D.M., Lauenborg J. (2009) Overweight and the metabolic syndrome in adult offspring of women with diet-treated gestational diabetes mellitus or type 1 diabetes. J. Clin. Endocrinol. Metab. 94, 2464–2470 19417040 10.1210/jc.2009-0305

[26] Norman R.J. and Clark A.M. (1998) Obesity and reproductive disorders: a review. Reprod. Fertil. Dev. 10, 55–63 9727593 10.1071/r98010

[27] Ryan D. (2007) Obesity in women: a life cycle of medical risk. Int. J. Obes. (Lond.) 31, S3–S7 17968435 10.1038/sj.ijo.0803729

[28] Jokela M., Elovainio M. and Kivimäki M. (2008) Lower fertility associated with obesity and underweight: the US National Longitudinal Survey of Youth. Am. J. Clin. Nutr. 88, 886–893 18842772 10.1093/ajcn/88.4.886

[29] van Nas A., Guhathakurta D., Wang S.S., Yehya N., Horvath S., Zhang B. (2009) Elucidating the role of gonadal hormones in sexually dimorphic gene coexpression networks. Endocrinology 150, 1235–1249 18974276 10.1210/en.2008-0563 PMC2654741

[30] Randall J.C., Winkler T.W., Kutalik Z., Berndt S.I., Jackson A.U., Monda K.L. (2013) Sex-stratified genome-wide association studies including 270,000 individuals show sexual dimorphism in genetic loci for anthropometric traits. PLoS Genet. 9, e1003500 23754948 10.1371/journal.pgen.1003500 PMC3674993

[31] Gesta S., Blüher M., Yamamoto Y., Norris A.W., Berndt J., Kralisch S. (2006) Evidence for a role of developmental genes in the origin of obesity and body fat distribution. Proc. Natl. Acad. Sci. U.S.A. 103, 6676–6681 16617105 10.1073/pnas.0601752103 PMC1458940

[32] Grove K.L., Fried S.K., Greenberg A.S., Xiao X.Q. and Clegg D.J. (2010) A microarray analysis of sexual dimorphism of adipose tissues in high-fat-diet-induced obese mice. Int. J. Obes. (Lond.) 34, 989–1000 20157318 10.1038/ijo.2010.12 PMC3667412

[33] Taylor R.W., Grant A.M., Williams S.M. and Goulding A. (2010) Sex differences in regional body fat distribution from pre-to postpuberty. Obesity (Silver Spring) 18, 1410–1416 19893501 10.1038/oby.2009.399

[34] Wells J.C., Treleaven P. and Cole T.J. (2007) BMI compared with 3-dimensional body shape: the UK National Sizing Survey. Am. J. Clin. Nutr. 85, 419–425 17284738 10.1093/ajcn/85.2.419

[35] Jackson A.S., Stanforth P.R., Gagnon J., Rankinen T., Leon A.S., Rao D.C. (2002) The effect of sex, age and race on estimating percentage body fat from body mass index: The Heritage Family Study. Int. J. Obes. Relat. Metab. Disord. 26, 789–796 12037649 10.1038/sj.ijo.0802006

[36] McQuaid S.E., Manolopoulos K.N., Dennis A.L., Cheeseman J., Karpe F. and Frayn K.N. (2010) Development of an arterio-venous difference method to study the metabolic physiology of the femoral adipose tissue depot. Obesity (Silver Spring) 18, 1055–1058 20057374 10.1038/oby.2009.486

[37] Sidebottom A.C., Brown J.E. and Jacobs D.R. Jr (2001) Pregnancy-related changes in body fat. Eur. J. Obstet. Gynecol. Reprod. Biol. 94, 216–223 11165728 10.1016/s0301-2115(00)00329-8

[38] Lassek W.D. and Gaulin S.J. (2006) Changes in body fat distribution in relation to parity in American women: a covert form of maternal depletion. Am. J. Phys. Anthropol. 131, 295–302 16596596 10.1002/ajpa.20394

[39] Pi-Sunyer F.X. (2004) The epidemiology of central fat distribution in relation to disease. Nutr. Rev. 62, S120–S126 15387477 10.1111/j.1753-4887.2004.tb00081.x

[40] Bouchard C., Pérusse L., Leblanc C., Tremblay A. and Thériault G. (1988) Inheritance of the amount and distribution of human body fat. Int. J. Obes. 12, 205–215 3391737

[41] Selby J.V., Newman B., Quesenberry C.P. Jr, Fabsitz R.R., Carmelli D., Meaney F.J. (1990) Genetic and behavioral influences on body fat distribution. Int. J. Obes. 14, 593–602 2228394

[42] Elks C.E., den Hoed M., Zhao J.H., Sharp S.J., Wareham N.J., Loos R.J. (2012) Variability in the heritability of body mass index: a systematic review and meta-regression. Front. Endocrinol. (Lausanne) 3, 29 22645519 10.3389/fendo.2012.00029 PMC3355836

[43] van Dongen J., Willemsen G., Chen W.-M., de Geus E.J. and Boomsma D.I. (2013) Heritability of metabolic syndrome traits in a large population-based sample. J. Lipid Res. 54, 2914–2923 23918046 10.1194/jlr.P041673 PMC3770104

[44] Rose K.M., Newman B., Mayer-Davis E.J. and Selby J.V. (1998) Genetic and behavioral determinants of waist-hip ratio and waist circumference in women twins. Obes. Res. 6, 383–392 9845227 10.1002/j.1550-8528.1998.tb00369.x

[45] Tenesa A. and Haley C.S. (2013) The heritability of human disease: estimation, uses and abuses. Nat. Rev. Genet. 14, 139–149 23329114 10.1038/nrg3377

[46] Rantalainen M., Herrera B.M., Nicholson G., Bowden R., Wills Q.F., Min J.L. (2011) MicroRNA expression in abdominal and gluteal adipose tissue is associated with mRNA expression levels and partly genetically driven. PLoS ONE 6, e27338 22102887 10.1371/journal.pone.0027338 PMC3216936

[47] Min J.L., Nicholson G., Halgrimsdottir I., Almstrup K., Petri A., Barrett A. (2012) Coexpression network analysis in abdominal and gluteal adipose tissue reveals regulatory genetic loci for metabolic syndrome and related phenotypes. PLoS Genet. 8, e1002505 22383892 10.1371/journal.pgen.1002505 PMC3285582

[48] Welter D., MacArthur J., Morales J., Burdett T., Hall P., Junkins H. (2014) The NHGRI GWAS Catalog, a curated resource of SNP-trait associations. Nucleic Acids Res. 42, D1001–D1006 24316577 10.1093/nar/gkt1229 PMC3965119

[49] Speliotes E.K., Willer C.J., Berndt S.I., Monda K.L., Thorleifsson G., Jackson A.U. (2010) Association analyses of 249,796 individuals reveal 18 new loci associated with body mass index. Nat. Genet. 42, 937–948 20935630 10.1038/ng.686 PMC3014648

[50] Willer C.J., Speliotes E.K., Loos R.J.F., Li S., Lindgren C.M., Heid I.M. (2009) Six new loci associated with body mass index highlight a neuronal influence on body weight regulation. Nat. Genet. 41, 25–34 19079261 10.1038/ng.287 PMC2695662

[51] Yang J., Loos R.J.F., Powell J.E., Medland S.E., Speliotes E.K., Chasman D.I. (2012) FTO genotype is associated with phenotypic variability of body mass index. Nature 490, 267–272 22982992 10.1038/nature11401 PMC3564953

[52] Lu Y., Day F.R., Gustafsson S., Buchkovich M.L., Na J., Bataille V. (2016) New loci for body fat percentage reveal link between adiposity and cardiometabolic disease risk. Nat. Commun. 7, 10495 26833246 10.1038/ncomms10495 PMC4740398

[53] Loos R.J.F., Lindgren C.M., Li S., Wheeler E., Zhao J.H., Prokopenko I. (2008) Common variants near MC4R are associated with fat mass, weight and risk of obesity. Nat. Genet. 40, 768–775 18454148 10.1038/ng.140 PMC2669167

[54] Teslovich T.M., Musunuru K., Smith A.V., Edmondson A.C., Stylianou I.M., Koseki M. (2010) Biological, clinical and population relevance of 95 loci for blood lipids. Nature 466, 707–713 20686565 10.1038/nature09270 PMC3039276

[55] Morris A.P., Voight B.F., Teslovich T.M., Ferreira T., Segrè A.V., Steinthorsdottir V. (2012) Large-scale association analysis provides insights into the genetic architecture and pathophysiology of type 2 diabetes. Nat. Genet. 44, 981–990 22885922 10.1038/ng.2383 PMC3442244

[56] Visscher P.M., Brown M.A., McCarthy M.I. and Yang J. (2012) Five years of GWAS discovery. Am. J. Hum. Genet. 90, 7–24 22243964 10.1016/j.ajhg.2011.11.029 PMC3257326

[57] Cariou B., Capitaine N., Le Marcis V., Vega N., Béréziat V., Kergoat M. (2004) Increased adipose tissue expression of Grb14 in several models of insulin resistance. FASEB J. 18, 965–967 15059968 10.1096/fj.03-0824fje

[58] Cooney G.J., Lyons R.J., Crew A.J., Jensen T.E., Molero J.C., Mitchell C.J. (2004) Improved glucose homeostasis and enhanced insulin signalling in Grb14-deficient mice. EMBO J. 23, 582–593 14749734 10.1038/sj.emboj.7600082 PMC1271812

[59] Clark M.E., Kelner G.S., Turbeville L.A., Boyer A., Arden K.C. and Maki R.A. (2000) ADAMTS9, a novel member of the ADAM-TS/ metallospondin gene family. Genomics 67, 343–350 10936055 10.1006/geno.2000.6246

[60] International HapMap Consortium, Frazer K.A., Ballinger D.G., Cox D.R., Hinds D.A., Stuve L.L. (2007) A second generation human haplotype map of over 3.1 million SNPs. Nature 449, 851–861 17943122 10.1038/nature06258 PMC2689609

[61] Mahajan A., Go M.J., Zhang W., Below J.E., Gaulton K.J., Ferreira T. (2014) Genome-wide trans-ancestry meta-analysis provides insight into the genetic architecture of type 2 diabetes susceptibility. Nat. Genet. 46, 234–244 24509480 10.1038/ng.2897 PMC3969612

[62] Savage D.B., Agostini M., Barroso I., Gurnell M., Luan J., Meirhaeghe A. (2002) Digenic inheritance of severe insulin resistance in a human pedigree. Nat. Genet. 31, 379–384 12118251 10.1038/ng926

[63] Choi J.H., Banks A.S., Estall J.L., Kajimura S., Boström P., Laznik D. (2010) Anti-diabetic drugs inhibit obesity-linked phosphorylation of PPARgamma by Cdk5. Nature 466, 451–456 20651683 10.1038/nature09291 PMC2987584

[64] Arnetz L., Dorkhan M., Alvarsson M., Brismar K. and Ekberg N.R. (2014) Gender differences in non-glycemic responses to improved insulin sensitivity by pioglitazone treatment in patients with type 2 diabetes. Acta Diabetol. 51, 185–192 23389468 10.1007/s00592-013-0457-y

[65] Chapman J.M., Cooper J.D., Todd J.A. and Clayton D.G. (2003) Detecting disease associations due to Llnkage disequilibrium using haplotype tags: a class of tests and the determinants of statistical power. Hum. Hered. 56, 18–31 14614235 10.1159/000073729

[66] Sudlow C., Gallacher J., Allen N., Beral V., Burton P., Danesh J. (2015) UK biobank: an open access resource for identifying the causes of a wide range of complex diseases of middle and old age. PLoS Med. 12, e1001779 25826379 10.1371/journal.pmed.1001779 PMC4380465

[67] Wood A.R., Esko T., Yang J., Vedantam S., Pers T.H., Gustafsson S. (2014) Defining the role of common variation in the genomic and biological architecture of adult human height. Nat. Genet. 46, 1173–1186 25282103 10.1038/ng.3097 PMC4250049

[68] Kichaev G. and Pasaniuc B. (2015) Leveraging functional-annotation data in trans-ethnic fine-mapping studies. Am. J. Hum. Genet. 97, 260–271 26189819 10.1016/j.ajhg.2015.06.007 PMC4573268

[69] Gaulton K.J., Ferreira T., Lee Y., Raimondo A., Mägi R., Reschen M.E. (2015) Genetic fine mapping and genomic annotation defines causal mechanisms at type 2 diabetes susceptibility loci. Nat. Genet. 47, 1415–1425 26551672 10.1038/ng.3437 PMC4666734

[70] Wellcome Trust Case Control Consortium, Maller J.B., McVean G., Byrnes J., Vukcevic D., Palin K. (2012) Bayesian refinement of association signals for 14 loci in 3 common diseases. Nat. Genet. 44, 1294–1301 23104008 10.1038/ng.2435 PMC3791416

[71] Lonsdale J., Thomas J., Salvatore M., Phillips R., Lo E., Shad S. (2013) The Genotype-Tissue Expression (GTEx) project. Nat. Genet. 45, 580–585 23715323 10.1038/ng.2653 PMC4010069

[72] Corradin O., Cohen A.J., Luppino J.M., Bayles I.M., Schumacher F.R. and Scacheri P.C. (2016) Modeling disease risk through analysis of physical interactions between genetic variants within chromatin regulatory circuitry. Nat. Genet., 48, 1313–1320 27643537 10.1038/ng.3674 PMC5083135

[73] Ioannidis N.M., Rothstein J.H., Pejaver V., Middha S., McDonnell S.K., Baheti S. (2016) REVEL: an ensemble method for predicting the pathogenicity of rare missense variants. Am. J. Hum. Genet., 99, 877–885 27666373 10.1016/j.ajhg.2016.08.016 PMC5065685

[74] Ionita-Laza I., McCallum K., Xu B. and Buxbaum J.D. (2016) A spectral approach integrating functional genomic annotations for coding and noncoding variants. Nat. Genet., 48, 214–220 26727659 10.1038/ng.3477 PMC4731313

[75] Ritchie G.R., Dunham I., Zeggini E. and Flicek P. (2014) Functional annotation of noncoding sequence variants. Nat. Methods, 11, 294–296 24487584 10.1038/nmeth.2832 PMC5015703

[76] McCarthy S., Das S., Kretzschmar W., Delaneau O., Wood A.R., Teumer A. (2016) A reference panel of 64,976 haplotypes for genotype imputation. Nat. Genet. 48, 1279–1283 27548312 10.1038/ng.3643 PMC5388176

[77] Gawad C., Koh W. and Quake S.R. (2016) Single-cell genome sequencing: current state of the science. Nat. Rev. Genet. 17, 175–188 26806412 10.1038/nrg.2015.16

[78] GTEx Consortium (2015) The Genotype-Tissue Expression (GTEx) pilot analysis: multitissue gene regulation in humans. Science 348, 648–660 25954001 10.1126/science.1262110 PMC4547484

[79] Soneson C. and Delorenzi M. (2013) A comparison of methods for differential expression analysis of RNA-seq data. BMC Bioinformatics 14, 91 23497356 10.1186/1471-2105-14-91 PMC3608160

[80] Hsu P.D., Lander E.S. and Zhang F. (2014) Development and applications of CRISPR-Cas9 for genome engineering. Cell 157, 1262–1278 24906146 10.1016/j.cell.2014.05.010 PMC4343198

[81] Witkos M., Uttaburanont M., Lang C.D. and Arora R. (2008) Costs of and reasons for obesity. J. Cardiometab. Syndr. 3, 173–176 18983335 10.1111/j.1559-4572.2008.00012.x

[82] Withrow D. and Alter D.A. (2011) The economic burden of obesity worldwide: a systematic review of the direct costs of obesity. Obes. Rev. 12, 131–141 20122135 10.1111/j.1467-789X.2009.00712.x

[83] Costello J.T., Bieuzen F. and Bleakley C.M. (2014) Where are all the female participants in Sports and Exercise Medicine research? Eur. J. Sport Sci. 14, 847–851 24766579 10.1080/17461391.2014.911354

[84] Mokdad A.H., Ford E.S., Bowman B.A., Dietz W.H., Vinicor F., Bales V.S. (2003) Prevalence of obesity, diabetes, and obesity-related health risk factors, 2001. JAMA 289, 76–79 12503980 10.1001/jama.289.1.76

